# Promoting exercise behavior and cardiorespiratory fitness among college students based on the motivation theory

**DOI:** 10.1186/s12889-022-13159-z

**Published:** 2022-04-13

**Authors:** Bo Li, Shanshan Han, Shuqiao Meng, Jaewoo Lee, Jie Cheng, Yang Liu

**Affiliations:** 1 Institute of Sports Science, Nantong University, Nantong, 226019 China; 2 School of Physical Education and Sport Training, Shanghai University of Sport, 650 Qing yuan huan Road, Yang Pu District, Shanghai, 200438 China; 3 Physical Education College of Yangzhou University, Yangzhou, 225127 China; 4 Kyunggi University, Graduate School, Suwon, Korea 16227 South Korea; 5 Physical Education College of Shanghai University, Shanghai, 200444 China; 6 Shanghai Research Center for Students Physique Health, Shanghai, 200438 China

**Keywords:** College students, Physical fitness indicator, Cardiovascular fitness, Motivation theory, Mediation effect

## Abstract

**Background:**

In recent years, there has been an increase in the incidence of health problems among college students in China, the lack of adequate physical exercise is the major reason. This study aimed to investigate methods to promote exercise behavior and cardiovascular fitness among college students based on the motivation theory.

**Methods:**

Cardiovascular fitness levels of 641 college students (20.72 ± 1.41 years old) were measured. Exercise motivation was assessed using the physical exercise motivation scale and physical exercise rating scale. Correlation analysis, regression analysis, and structural equation models were used to assess exercise motivation, exercise behavior, and cardiovascular fitness. Energy relationships were determined to develop a path model that promotes exercise behavior and aerobic fitness among college students.

**Results:**

The exercise motivation of college students was directly related to cardiovascular fitness (effect value: 0.577) or indirectly related through the mediating effect of exercise behavior (effect value: 0.215). The influence of health motivation on exercise behavior (β = 0.132, *p* = 0.001) and cardiovascular fitness (β = 0.251, *p* < 0.001) was greater than that of other factors of motivation.

**Conclusions:**

The physical exercise behavior of college students partially mediates the relationship between exercise motivation and cardiovascular fitness. Therefore, the educational concept of “Health First” should be promoted in college sports. Internal motivation of exercise can be transformed into external motivation to improve students’ exercise behavior and cardiovascular fitness through enhancing their cardiopulmonary capacity.

## Background

The college student population is a reserve of high-quality national talent. However, the decline in the health of college students is of great concern. From 1985 to 2014, 1,513,435 students participated in the Chinese National Survey on Student Constitution and Health. A decline in the physical fitness indicator (PFI) was observed between 1985 and 2014 (overall PFI change of 0.8), albeit with an increase from 1985 to 1995 (PFI change of 1.2). This coincides with a shift in major nutritional problems from the stunting and thinness stages to the overweight and obesity stages. Both undernourished (PFI of − 2.44 for thin and − 3.42 for stunting) and overnourished (− 1.49 for overweight and − 3.63 for obese) students had lower PFIs than those with weight in the normal range (− 0.41) in 2014 [1]. Wu et al. arrived at the same conclusion [2]. This confirms the decline in the physical fitness of college students.

The lack of adequate physical exercise is one of the major reasons for the health problems of college students. In 2016, the 27th document issued by the State Council was the “*Opinions of the General Office of the State Council on Strengthening School Sports to Promote the Comprehensive Development of Students’ Physical and Mental Health.*” This document highlighted that the physical fitness of students remains a shortcoming of their physical and mental health. Document No. 27 proposed several measures to improve students’ physiques, which reflects the significant attention that the Party Central Committee directed toward the health of students’ physiques. Hence, it is necessary to promote studies focusing on paths of students’ physical behavior. In recent years, scholars have achieved promising results with respect to investigation of methods to promote exercise behavior. In numerous studies, the improvement path of student physiques was investigated based on various theories such as the self-determination theory [3, 4], planned behavior theory [5], and health behavioral process theory [6], of which, the relevant achievements of the motivation theory are clear [7]. The core of the motivation theory is the causes of certain behaviors, and its premise assumes that there are causes for the occurrence of these behaviors [8]. Motivation for physical exercise refers to the psychological motivation that promotes physical exercise [9]. The investigation of the physical problems of college students based on the motivation theory serves as a reliable theoretical basis.

Previous studies have revealed that the coexistence of internal and external motivations for physical exercise can better promote physical exercise and motivate individuals to engage in physical exercise, which has a significant influence on the emotional experiences and mental health of individuals [10–12]. Studies conducted by Liu have revealed that the motivation for outdoor sports in adolescents can significantly predict the persistence of exercise behavior [13], which has also been verified in previous studies [14]. The relationship between motivation and exercise behavior supports the motivation theory. Moreover, exercise time, intensity, frequency, and amount exhibit a significant positive correlation [4]. The student PFI is a measure of cardiovascular fitness, which is an important branch of student physical fitness. Cardiovascular fitness is an important indicator for student fitness monitoring via fitness tests in various countries worldwide. In China, the test indicators for the cardiovascular fitness of college students are based on the time required to run distances of 1000 m and 800 m for boys and girls, respectively.

The high correlation between the motivation of college students’ exercise participation and healthy physical fitness has been verified by most empirical studies [15–17]. Whether it is behavioral or epidemiological research scholars, they usually focus on changing the sports participation behavior of subjects by improving the motivation of sports participation in different groups, including increasing the frequency of exercise participation, increasing the intensity of exercise or/ and increasing the duration of exercise. The ultimate goal of these changes is to improve people’s health and the performance variable is generally healthy physical fitness [18].

In the present study, the research on cardiovascular fitness was mainly based on the following. (1) Cardiovascular fitness can reflect the ability of the human cardiovascular system, and forms the basis of qualities such as strength and endurance. Good cardiovascular fitness is an important factor with respect to various chronic diseases. (2) The main form of exercise for cardiovascular fitness is “*continuous aerobic exercise*” [19], and continuous cardiovascular fitness is the most essential exercise method for college students, which was verified in the questionnaire survey of this study. It should be noted that, in this study, 74.5% of college students selected continuous aerobic exercise such as jogging, yoga, and Pilates, among which, jogging was the most frequent exercise method. Among the influencing factors of cardiovascular fitness, previous research has revealed that for different age groups, physical activity is among the main constraints to cardiovascular fitness [20, 21]. Moreover, a correlation was confirmed between physical exercise behavior and cardiovascular fitness.

**Fig. 1:**
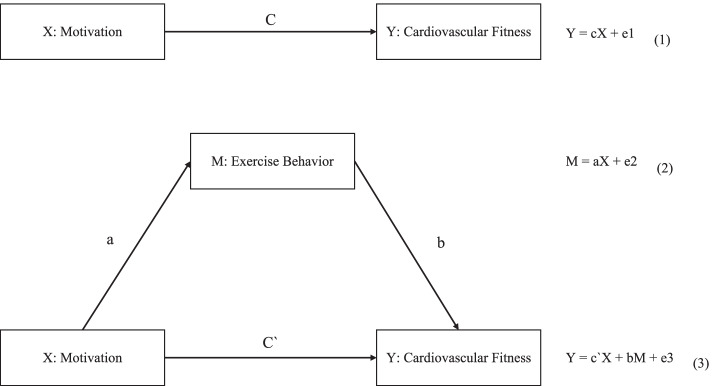
The conceptual model of the mediation effect

In previous research, Yang investigated methods to promote cardiovascular fitness and exercise behavior among adolescents based on the motivation theory. The results revealed that exercise behavior plays a partial mediating role between exercise motivation and cardiovascular fitness of adolescents [22], which serves as a theoretical basis for the establishment of a conceptual model of intermediary effects. However, we decided that the majority of survey subjects in our study would be children and adolescents, and the discussion on related variables would be based on the relationship between the two variables. Hence, we attempted to develop a motivational path model that promotes the cardiovascular fitness and exercise behavior of college students. According to the Antecedents → Behavioral process → Consequences research ideas commonly used in organizational behavior, students’ exercise motivation and cardiovascular fitness are used as the antecedent and outcome variables, respectively, to investigate their exercise motivation, exercise behavior, and cardiovascular fitness. We also considered the mediating effects of relationship and exercise behavior. Therefore, the research hypotheses (H1–H4) were as follows (Fig. 1):

H1: Exercise motivation can significantly and positively predict physical exercise behavior.

H2: Exercise motivation can significantly predict cardiovascular fitness.

H3: Exercise behavior can significantly and positively predict cardiovascular fitness.

H4: Exercise motivation can indirectly influence cardiovascular fitness through an intermediary of exercise behavior.

Based on these hypotheses, this study aimed to investigate methods to promote exercise behavior and cardiovascular fitness among college students based on the motivation theory.

## Methods

We investigated methods to promote exercise behavior and cardiovascular fitness among college students based on the motivation theory. This research adopted a cross-sectional research design, the key element of which was the participation of physical education teachers in the research implementation. After the research plan was formulated, three “university physical education” course teachers from three schools A, B, and C were recruited for a fee, and three teachers were tested. All the tests in this study were completed in the “University Physical Education” course, which is a nationally prescribed curriculum for Chinese college students. Recruitment for the test was conducted among freshmen and sophomores from three universities in Shanghai. The main exposure indicators included: sociodemographic information (gender, age, grade), exercise behavior (duration, frequency, intensity, and exercise items), exercise motivation, and cardiovascular fitness. The entire test started on March 1, 2018 and ended on April 30, 2018. The total duration lasted 43 working days. All methods were carried out in accordance with relevant guidelines and regulations.

### Participants

Using cluster sampling, questionnaires and cardiovascular fitness tests were conducted on freshmen and sophomores at three universities in Shanghai in March and April 2018. The study was based on the National Student’s Body Health Standards set by the Chinese Ministry of Education, which waived the requirement for obtaining ethical approval for the test’s design. Before formal investigation and testing, the researchers obtained the informed consent of the subjects involved in this study. Students with major organic diseases (e.g., heart, lung, liver, kidney, and related diseases), abnormal body development, or cause-based obesity (e.g., endocrine disease or drug side effect) were excluded. The sample size of the questionnaire data was 1049, and the sample size of the cardiovascular fitness data was 781. The two data points were matched using the numbers of students. The sample size of the matching data was 641 (317 males, accounting for 49.45%, and 324 females, accounting for 50.55%).

### Evaluation instruments

#### Physical activity rating scale

The physical exercise rating scale was compiled by the Japanese scholar Takao Hashimoto, which was subsequently introduced and completed by Liang in China. Physical exercise volume was examined with respect to the exercise intensity, frequency, and time of one exercise activity to measure physical exercise participation [23].

#### Physical exercise volume score = intensity × (time – 1) × frequency

Each parameter was evaluated using five score levels. The level standards were as follows: small exercise volume ≤ 19 points, moderate exercise volume = 20–42 points, and large exercise amount ≥ 43 points [23]. The re-test reliability of this scale was 0.820. Follow-up related research shows that the internal consistency reliability of PARS-3 is Cronbach’s α = 0.85. The value of the scale was used to reflect the physical exercise behaviors of college students.

#### Motives for physical activities measure-revised (MPAM-R)

The MPAM-R compiled by Ryan et al. is a commonly used tool for determining exercise motivation [24]. This exercise motivation scale is based on the cognitive evaluation theory and self-determination theory, which includes five motivation dimensions. In particular, external motivation includes the promotion of health (Fitness) and improvement of appearance (Appearance), and internal motivation includes fun (Enjoyment), improvement of ability (Competence), and socializing (Social) [11]. The Chinese version of the scale is adequately reliable and valid. Cronbach’s ɑ of the total scale was 0.922, and the reliability coefficients of each subscale ranged from 0.81 to 0.91. The calibration correlation validity was used to verify exercise motivation and exercise. The results of the behavioral relationship investigation have revealed that exercise motivation, frequency, duration, and intensity exhibit a significant positive correlation, indicating that the validity of the scale is high [25]. To simply measure exercise motivation, Chen et al. reduced the Chinese version of the MPAM-R to a 15-item simplified scale [25]. In this study, a simplified version of the motivation scale was used.

### Cardiovascular fitness test

The cardiovascular fitness test was conducted in accordance with the relevant requirements of *the National Student Physical Health Standard (Revised in 2014)***(**Table 1**)**. The test items were time required to run distances of 1000 m for boys and 800 m for girls. The scoring method also used standard requirements. During the field test, the time required for the subjects to run the 1000 m/800 m at full strength was recorded, and the time was assigned during the statistical analysis. The comparison standard is shown in Table 1. During the aerobic fitness test, the students completed a questionnaire.

**Table 1:** Cardiovascular fitness test scoring table

Gender Score	Male		Female	
	1–2	3–4	1–2	3–4
100	3′17”	3′15”	3′18”	3′16”
95	3′22”	3′20”	3′24”	3′22”
90	3′27”	3′25”	3′30”	3′28”
85	3′34”	3′32”	3′37”	3′35”
80	3′42”	3′40”	3′44”	3′42”
78	3′47”	3′45”	3′49”	3′47”
76	3′52”	3′50”	3′54”	3′52”
74	3′57”	3′55”	3′59”	3′57”
72	4′02”	4′00”	4′04”	4′02”
70	4′07”	4′05”	4′09”	4′07”
68	4′12”	4′10”	4′14”	4′12”
66	4′17”	4′15”	4′19”	4′17”
64	4′22”	4′20”	4′24”	4′22”
62	4′27”	4′25”	4′29”	4′27”
60	4′32”	4′30”	4′34”	4′32”
50	4′52”	4′50”	4′44”	4′42”
40	5′12”	5′10”	4′54”	4′52”
30	5′32”	5′30”	5′04”	5′02”
20	5′52”	5′50”	5′14”	5′12”
10	6′12”	6′10”	5′24”	5′22”

*Note*. Data sourced from National Student Physical Health Standard (Revised in 2014)

**Table 2:** Normal distribution test table

	Cardiorespiratory fitness	Physical exercise	Motivation
Skewness	−0.456	1.265	−1.597
Std. Error of Skewness	0.097	0.097	0.097
Z-Score	−4.725	13.102	−16.550
Kurtosis	0.006	1.735	4.473
Std. Error of Kurtosis	0.193	0.193	0.193
Z-Score	0.033	9.000	23.204

Skewness and kurtosis are used for normality test, and its corresponding Z-score is calculated, namely: skewness Z-score = skewness value/standard error, kurtosis Z-score = kurtosis value/standard error. At the test level of α = 0.05, if the Z-score is between ±1.96, the data can be considered to obey a normal distribution. The SPSS25.0 software is used to test the normality of cardiorespiratory fitness, physical exercise and motivation. The results are shown in the Table 2. The data shows that the above indicators do not follow a normal distribution.

The cardiovascular fitness test was performed as part of the college physical education curriculum. Before the test, students needed to perform a standardized warm-up for 5 min, including muscle stretching and joint exercises. According to the curriculum arrangement of the test school, college physical education is generally arranged between 9:00–11:00 in the morning and 2:00–5:00 in the afternoon, and the physical education class is only available under clear and conducive weather and temperature conditions. The test was conducted in the physical education class, enabling suitable temperature and humidity during the test.

Cardiovascular fitness was carried out in accordance with the test items of China’s “National Student Physical Fitness and Health Standard”, which is a national behavior. The specific standardized test procedures for the test items of the “National Standards for Students’ Physical Fitness and Health” for the 1000 m or 800 m race are as follows.

① Test purpose. Test the development level of students’ endurance quality, especially the function of the cardiovascular system and muscle endurance.

② Test equipment. 400 m, 300 m, 200 m track and field runways, geology is not limited. Other irregular venues can also be used, but the measurement must be accurate and the venue flat. Several stopwatches need to be corrected before use, and the error per min should not exceed 0.2 s. The standard stopwatch is selected based on Beijing time, with an error of no more than 0.3 s per h.

③ Test method. At least two subjects will be tested in pairs, commencing from a standing start point. Start running after hearing the “Run” command. The timekeeper will see the flag to start the watch, and stop the watch when the subject’s torso reaches the vertical plane of the finish line. Record the test results in seconds, accurate to one digit after the decimal point, and the second digit after the decimal point is considered according to the principle of non-zero advancement: for example, 10.11 s as 10.2 s.

④ Matters needing attention. A. It is best for subjects to wear sports shoes or flat shoes during the test. Spikes, leather shoes, and plastic sandals are not allowed. B. If a runner is found, it should be recalled immediately and run again. C. Run with the wind if encountering it in the course of the run.

### Common method deviation test

Program control and Harman’s single-factor test were used to control the common method of deviation [26]. With respect to program control, classic measuring tools were used after language system localization and reliability and validity monitoring. Moreover, the questionnaires were designed with bold font and emphasis on annotations. During the questionnaire filling process, it was emphasized that “*the survey was only for scientific research, and the results of the questionnaire were only for the researcher.*” The surveyor was a physical education teacher who collected data in real-time by answering questions during exercise and recovery. With respect to Harman’s single-factor test, a single-factor unrotated exploratory factor analysis was performed on all items, with the exception of demographic variables.

### Statistical analysis

The IBM SPSS Statistics 23.0 software and process macro programs were used for data processing. To simplify the analysis, demographic variables such as gender and age are not described separately in the remainder of this paper. (1) Correlation analysis was conducted to investigate the correlation among exercise motivation, behavior, and cardiovascular fitness. (2) Linear regression was used to investigate the predictive strength of exercise motivation on exercise behavior and cardiovascular fitness and the predictive strength of exercise behavior on cardiovascular fitness. (3) Based on a study conducted by Fang and Zhang [27], the bootstrap method was used to analyze the indirect influence of exercise motivation on cardiovascular fitness. Moreover, PROCESS Model 4 was used to test the intermediary effect [28], and the independent variable (X), dependent variable (Y), and the intermediary variable (M) were sequentially selected in the corresponding option box. The bootstrap sample size was set as 5000, the sampling method was corrected for bias (i.e., the non-parameter percentile method for deviation correction), and the confidence interval was 95%. In addition, the grouping condition was mean and mean ± standard deviation (SD), and the test level was α = 0.05.

## Results

### Common method deviation test

As a result of the common method deviation test, six factors with feature root values greater than 1 were extracted, and the largest factor variance was 23.42%. Less than 40% of the standard critical value. It was confirmed that the common method deviation was not significant.

### Fundamental cases and related analysis of exercise motivation, behavior, and cardiovascular fitness

Table 3 shows that the average value of each dimension of exercise motivation is health, appearance, ability, and socializing in order from high to low and that the score of external motivation was significantly higher than that of internal motivation. Pearson’s correlation analysis results revealed that there is no correlation between exercise behavior and social motivation. Aerobic fitness exhibited no correlation with exercise motivation, social motivation, and exercise duration. Motivation was significantly related to cardiovascular fitness and physical exercise behavior (*p* < 0.05). Moreover, a high degree of correlation was found among various dimensions of motivation.

**Table 3:** Pearson’s bivariate bilateral correlation coefficient table

	M	SD	1	1–1	1–2	1–3	1–4	1–5	2	2–1	2–2	2–3
**1 Motivation**	20.43	3.86										
**1–1 Health**	4.25	0.834	0.903^**^									
			0.887,0.916									
**1–2 Appearance**	4.21	0.874	0.867^**^	0.814^**^								
			0.846,0.885	0.786,0.839								
**1–3 Pleasure**	4.09	0.882	0.912^**^	0.826^**^	0.772^**^							
			0.898, 0.924	0.800,0.849	0.739,0.801							
**1–4 Ability**	4.1	0.917	0.834^**^	0.661^**^	0.596^**^	0.671^**^						
			0.808,0.856	0.615,0.702	0.543,0.643	0.626,0.711						
**1–5 Social**	3.89	0.992	0.846^**^	0.655^**^	0.612^**^	0.715^**^	0.691^**^					
			0.823,0.867	0.607,0.696	0.561,0.658	0.674,0.750	0.642,0.729					
**2 Exercise behavior**	21.25	17.693	0.112^**^	0.087^*^	0.093^*^	0.103^**^	0.132^**^	0.074				
			0.035,0.187	0.011,0.164	0.016,0.169	0.026,0.179	0.056,0.208	−0.004,0.149				
**2–1 Strength**	2.47	1.192	0.089^*^	0.072	0.051	0.074	0.123^**^	0.066	0.783^**^			
			0.012,0.165	−0.005,0.148	−0.026,0.128	− 0.003,0.150	0.046,0.199	− 0.011,0.142	0.751,0.811			
**2–2 Duration**	3.47	1.097	0.131^**^	0.091^*^	0.114^**^	0.151^**^	0.140^**^	0.075	0.728^**^	0.420^**^		
			0.055,0.206	0.015,0.168	0.038,0.190	0.075,0.226	0.063,0.214	−0.002,0.151	0.690,0.763	0.354,0.482		
**2–3 Frequency**	3.12	0.818	0.085^*^	0.06	0.103^**^	0.043	0.080^*^	0.084^*^	0.351^**^	0.042	0.149^**^	
			0.008, 0.161	−0.018,0.136	0.026,0.179	−0.034,0.120	0.003,0.156	0.007,0.160	0.281,0.416	−0.035,0.119	0.072,0.223	
**3 Cardiovascular fitness**	66.3	12.09	0.324^**^	0.275^**^	0.252^**^	0.078	0.324^**^	0.187	0.248^**^	0.200^*^	0.174	0.128^**^
			0.002,0.152	0.013,0.141	0.009,0.162	−0.020,0.134	0.041,0.193	−0.075,0.079	0.048,0.107	0.039,0.116	−0.059,0.095	0.007,0.160

*Note*. **Correlation is significant at a level of 0.01. *Correlation is significant at a level of 0.01

*M* mean, *SD* standard deviation

### Regression analysis of exercise motivation, behavior, and cardiovascular fitness

The variance test in the regression analysis revealed that all the ***p*-**values were less than 0.05, and the regression model was effective (Table 4). The regression results of Eq. (1) in Table 3 revealed that exercise motivation can significantly predict aerobic fitness (F = 75.057, *p* < 0.001), which explains 10.5% of the variations in aerobic fitness. The regression results of Eq. (2) in Table 3 revealed that exercise motivation can significantly and positively predict physical exercise behavior (F = 8.814, *p* = 0.040), which explains 1.3% of the variations in exercise behavior. The regression results of Eq. (3) in Table 3 revealed that the independent variables include exercise motivation and behavior, which explain 15.1% of the variations in cardiovascular fitness (F = 56.554, *p* < 0.001). Overall, exercise motivation and behavior can significantly predict cardiovascular fitness. From Eq. (1), it was found that exercise motivation is the cause of 10.4% of the variations in cardiovascular fitness. When Eq. (3) sports participation behavior. After intervention, the variations in exercise motivation with respect to cardiovascular fitness increased to 15.1%, whereas the regression coefficient of exercise motivation with respect to cardiovascular fitness decreased from 0.324 (0.3 value), as expressed by Eq. (1), to 0.300 (β value). Exercise behavior can significantly predict cardiovascular fitness. Moreover, there was a partial mediation effect between exercise motivation and aerobic fitness. However, the value of the mediation effect requires further calculation, as discussed in the Construction and verification of structural relationship model section.

**Table 4:** Regression analysis index of each equation

	Model summary		ANOVA		Coefficients				
	R^2^	Adjusted R^2^	F	*p*	B	SE	β	t	*p*
Equation (1)	0.105	0.104	75.057	0.000	0.821	0.095	0.324	8.664	0.000
Equation (2)	0.013	0.011	8.814	0.040	0.515	0.180	0.112	2.861	0.004
Equation (3)	0.151	0.148	56.554	0.000	0.760	0.093	0.300	8.172	0.000
					0.119	0.020	0.215	5.844	0.000

*ANOVA* Analysis of variance, *SE* standard error

To further investigate the predictive strengths of different dimensions of exercise motivation on the behavior and cardiovascular fitness variables, the following process was conducted (Table 5). The stepwise regression method was employed for analysis; 0.1 was inputted into the “probability of using F” and the input 0.11 was deleted; that is, when the ***p*** value of the maximum ***F*** of the candidate variable was less than or equal to 0.1, the relevant variable was introduced. Among the variables, when the *p* value of the smallest F value was greater than or equal to 0.1, the variable was eliminated. The results of the stepwise regression analysis revealed that the equation yielded *p* < 0.05 and that the two regression models were effective. In the predictive exercise behavior model, the health motivation dimension was inputted into the model. In the predicted cardiovascular fitness model, the health and ability motivation dimensions were inputted into the model. In comparison, health motivation (β = 0.251) was found to be more significant than ability (β = 0.110), which exhibited a high prediction strength.

**Table 5:** Stepwise regression analysis index

	Exercise behavior				Cardiovascular fitness			
	β	*p*	R^2^	F	β		R^2^	F
Motivation			0.017	11.349**			0.109	40.114***
1–1 Health	0.132	0.001			0.251	0.000		
1–2 Appearance	Exclude				Exclude			
1–3 Pleasure	Exclude				Exclude			
1–4 Ability	Exclude				0.110	0.030		
1–5 Social	Exclude				Exclude			

*Note*. **p < 0.01; ***p < 0.001

### Construction and verification of the structural relationship model

Using PROCESS Model 4 (simple mediation model) in the SPSS macro, as compiled by Bolin [28], the mediation effect of exercise behavior between the exercise objective and cardiovascular fitness was investigated by controlling for adjustment variables such as gender and grade (Table 6). The results revealed the direct influence of exercise motivation on cardiovascular fitness and the mediating effect of exercise behavior. The bootstrap 95% upper and lower levels of confidence intervals did not include 0 in the upper and lower limits, which revealed that exercise motivation can directly predict cardiovascular fitness and aerobic fitness through the mediation of exercise behavior. Direct effects (0.557) and intermediary effects (0.251) accounted for 68.9 and 31.1% of the total effects (0.808), respectively.

**Table 6:** List of total effects, direct effects, and intermediary effects

	Effect	Boot SE	Boot LLCI	Boot ULCI	Value of opposite effect
Total effects	0.808	0.114	0.592	1.033	
Direct effects	0.557	0.110	0.551	0.978	68.9%
Indirect effects	0.251	0.023	0.010	0.102	31.1%

*SE* standard error, *LLCI* lower level of confidence interval, *ULCI* upper level of confidence interval

## Discussion

Our research explored the relationship between college students’ exercise motivation, exercise behavior and cardiorespiratory fitness through cross-sectional design. We found that the motivation of college students to exercise can directly affect aerobic fitness, and it can also indirectly affect aerobic fitness through the mediating effect of exercise behavior. Health motivation has greater influence on exercise behavior and aerobic fitness than other dimensions of motivation. A possible reason is the influence of the “Healthy China 2030” plan issued by the Chinese government. It is posited that people’s pursuit of health is much higher than other exercise motivations.

### Influence of exercise motivation on exercise behavior

The results of this study validate H1, i.e., exercise motivation can significantly predict exercise behavior. This is because the prediction strength of health motivation, which is a part of external motivation, was greater than that of the others. The results revealed that the amount of physical exercise can be increased by increasing the motivation to exercise among college students, followed by an increase in the amount of physical activity. Our results verify several conclusions reached in previous studies. In particular, Litt et al. studied the relationships between physical exercise motivation and physical exercise behavior of 9011 adolescents and found that external motivation (health) is more predictive of physical exercise behavior than internal motivation [29]. Our results further validate the motivation theory. One of the core objectives of the exercise motivation theory is to stimulate and encourage more people to exercise [30]. Moreover, our results revealed that among the different dimensions of motivation, health motivation exhibited a relatively high prediction strength.

As indicated by the descriptive analysis results, the motivation with respect to different dimensions from strong to weak prediction strength were as follows: health, appearance, ability, ability, and social. In comparison, the external motivation of college students to participate in physical exercise was stronger than the internal motivation. The results of a previous study on the motivation and exercise persistence of college students revealed that the health motivation of college students was the strongest, followed by fun and ability, whereas socializing and appearance motivation were the weakest [31]. Kilpatrick et al. found that the main motivation for female college students to exercise is to improve their physical appearance, whereas the main motivation for male college students is to improve their social interaction [15]. The results of the abovementioned studies are significantly different from the results of our study, which may be due to the influence of culture, atmosphere, and other factors on the generation of exercise motivation.

### Influence of exercise motivation on cardiovascular fitness

The results of this study validate H2 as well, that is, exercise motivation can significantly predict cardiovascular fitness. This is because the prediction strength of the physical and mental health motivation dimension (β = 0.251) and the skill promotion motivation dimension (β = 0.110) was the greatest. According to the structural model test results, the relative effect value of the direct influence of exercise motivation on cardiovascular fitness was 68.9%. These results indicate that an improvement in cardiovascular fitness can be promoted by enhancing the exercise motivation of college students. Moreover, the results of our study verify H3 and H4, that is, exercise behavior can significantly predict cardiovascular fitness and exercise motivation can indirectly influence cardiovascular fitness through the intermediary of exercise behavior, with a relative effect value of 31.1%. This indicates that in the context of physical exercise, exercise behavior has a partial mediating effect between the exercise motivation and cardiovascular fitness of college students.

A study conducted by Mcdavid et al. revealed that exercise motivation provided by physical education teachers and parents can increase the amount of children’s leisure-time physical activity [32]. In a study conducted by Wang, exercise motivation could predict the cardiovascular fitness levels of young students. However, among the five dimensions, only fun (β = 0.145), ability (β = 0.214), and socializing (β = 0.176) exhibited predictive values. The differences in the results can be attributed to differences in the age groups of the research subjects [33]. More studies have been conducted on exercise motivation and cardiovascular fitness in children and adolescents, whereas studies conducted on college students have focused on the influence of peer support, exercise confidence, exercise attitude, and other variables on the constitution of college students. Based on the analysis, this is because most college students are adults. The acquisition and promotion paths of exercise motivation are not as extensive for college students as those for children and adolescents. The exercise motivation of children and adolescents may originate from school, family, and the society. In comparison, the exercise motivation of college students is more dependent on their health consciousness, career outlook, and enrollment in college physical education courses. The motivation for improving exercise motivation was, therefore, low.

The data revealed that it is feasible to construct a path model to promote the cardiovascular fitness and exercise behavior of college students based on the motivation theory. The establishment of this model can provide a theoretical basis for the study of the physical fitness promotion paths of college students. Cardiovascular fitness is an important dimension of the physical fitness of students. Moreover, physical fitness is the major priority of physical education in schools. In the early 1980s, with the establishment of the idea of “physical education,” such education developed into an independent ideological system. The Chinese “*National Student Physical Health Test*,” as a national census, is an important evaluation method in school physical education. “*Enhancing student physical fitness*” is included in the “*School Physical Education Work Regulations*” as the fundamental task of school physical education. Therefore, the construction of a theoretical model is necessary. Moreover, in 1999, the “*Decision of the Central Committee of the Communist Party of China on Deepening Educational Reform and Promoting Quality Education in an All-round Way*” proposed that “*school sports should establish the guiding ideology of health first*.” The concept of “*Health First*” integrates the concepts of physical education and actual physical education practice. As indicated by the results of our study, health motivation as an external motivation ranks first among other dimensions of motivation with respect to the prediction of exercise behavior and cardiovascular fitness, which is partly guided by the “*Health First*” school sports objective.

The results of our study revealed that not all dimensions of motivation have significant predictive strengths; the predictive strength of external motivation was higher. Conceptually, internal motivation refers to participation in physical activities for the improvement of ability, fun, and socialization, whereas external motivation refers to participation in physical activities due to instrumental factors such as external pressure and rewards. In previous studies, it was suggested that external motivation weakens internal motivation and promotes internal motivation [34, 35]. Therefore, promoting the conversion of internal motivation to external motivation is a suitable method for the promotion of overall motivation. In addition, gender, age, and other demographic variables were controlled in our study. However, the results of previous studies have revealed that the relationship between exercise motivation and exercise volume and physical fitness is regulated, to an extent, by factors such as gender, school period, area, and school nature [36]. In addition, corresponding research in a cross-sectional study confirming whether it is a complete intermediary model or a partial intermediate decomposition model stated that there is a deviation between the direct and intermediary effects [37]. Therefore, follow-up studies should be conducted with an expanded sample size and an intervention study design.

However, our study has some limitations. First, our research is cross-sectional, thus causal inferences cannot be determined. This study only explored the relationship between variables through cross-sectional data. Although the mediation effect model was used to explore the influence paths between related variables, the causal relationship between variables could not be clearly analyzed. Second, due to limitations in research funding, we used mature scales for the measurement of physical exercise, and we did not use objective methods such as accelerometers, which will also affect the validity of the research conclusions to a certain extent. In our upcoming research, we will follow up the corresponding research results in time, design longitudinal data collection strategies and methods on the basis of this research, and analyze the strength and direction of the causal relationship of exercise motivation, exercise behavior, and cardiorespiratory fitness.

**Suggestions**

1. Continue to strengthen the educational concept of “*Health First*” in college sports and improve the motivation of college students to exercise.
2. In implementation of the college physical education curriculum, attention should be directed toward the transformation of the internal motivation of students to participate in physical exercise into external motivation to enhance their motivation for exercise.
3. Although motivation is an influence on exercise behavior and cardiovascular fitness, it is limited. Therefore, further studies including a larger sample size and expanding the research on other influencing factors of exercise behavior and cardiovascular fitness of college students are warranted.

## Conclusions

The exercise motivation of college students can directly influence cardiovascular fitness or indirectly influence cardiovascular fitness through the mediating effect of exercise behavior. The motivation path can be described as follows. (1) Exercise motivation can significantly and positively predict physical exercise behavior; (2) exercise motivation can significantly predict cardiovascular fitness; (3) exercise behavior can significantly and positively predict cardiovascular fitness; and (4) exercise motivation can indirectly influence cardiovascular fitness through the intermediary of exercise behavior.

Based on the corresponding evidence, it was found that by motivating college students to exercise, they can strengthen their exercise volume, improve their aerobic fitness levels, and ultimately promote their physical fitness. The influence of healthy motivation on exercise behavior and cardiovascular fitness was greater than that of other dimensions of motivation. Moreover, the establishment of the relationship among these factors requires the support of subsequent longitudinal data.

## Data Availability

The datasets used and/or analyzed during this survey are available from the corresponding author upon reasonable request.

## Abbreviations

PFI: Physical fitness indicator
MPAM-R: Motives for Physical Activities Measure-Revised
